# 16/6-idiotype expressing antibodies induce brain inflammation and cognitive impairment in mice: the mosaic of central nervous system involvement in lupus

**DOI:** 10.1186/1741-7015-11-90

**Published:** 2013-04-04

**Authors:** Shaye Kivity, Aviva Katzav, Maria Teresa Arango, Moran Landau-Rabi, Yaron Zafrir, Nancy Agmon-Levin, Miri Blank, Juan-Manuel Anaya, Edna Mozes, Joab Chapman, Yehuda Shoenfeld

**Affiliations:** 1 The Zabludovicz Center for Autoimmune Diseases, Sheba Medical Center, Tel-Hashomer, 2 Derech Sheba St., Ramat Gan, 52621, Israel; 2 Rheumatic Disease Unit, Sheba Medical Center, Tel-Hashomer, 2 Derech Sheba St., Ramat Gan, 52621, Israel; 3 Department of Neurology, Sagol Neuroscience Center, Sheba Medical Center, Tel-Hashomer, 2 Derech Sheba St., Ramat Gan, 52621, Israel; 4 Center for Autoimmune Diseases Research (CREA), School of Medicine and Health Sciences, Universidad del Rosario, Kr 24 N°63C-69, Bogotá, DC, 111221, Colombia; 5 Doctoral Program in Biomedical Sciences, Universidad del Rosario, Kr 24 N°63C-69, Bogotá, DC, 111221, Colombia; 6 Department of Immunology, The Weizmann Institute of Science, 234 Herzl Street, Rehovot, 76100, Israel; 7 Incumbent of the Laura Schwarz-Kip Chair for Research of Autoimmune Diseases, Sackler Faculty of Medicine, Tel-Aviv University, Haim Lebanon St., Ramat Aviv, 69978, Israel

**Keywords:** Systemic lupus erythematosus, 16/6 idiotype, Anti-DNA, Neuropsychiatric lupus, Cognitive impairment

## Abstract

**Background:**

The 16/6-idiotype (16/6-Id) of the human anti-DNA antibody was found to induce experimental lupus in naïve mice, manifested by production of autoantibodies, leukopenia and elevated inflammatory markers, as well as kidney and brain involvement. We assessed behavior and brain pathology of naive mice injected intra-cerebra-ventricularly (ICV) with the 16/6-Id antibody.

**Methods:**

C3H female mice were injected ICV to the right hemisphere with the human 16/6-Id antibody or commercial human IgG antibodies (control). The mice were tested for depression by the forced swimming test (FST), locomotor and explorative activity by the staircase test, and cognitive functions were examined by the novel object recognition and Y-maze tests. Brain slices were stained for inflammatory processes.

**Results:**

16/6-Id injected mice were cognitively impaired as shown by significant differences in the preference for a new object in the novel object recognition test compared to controls (*P* = 0.012). Similarly, the preference for spatial novelty in the Y-maze test was significantly higher in the control group compared to the 16/6-Id-injected mice (42% vs. 9%, respectively, *P* = 0.065). Depression–like behavior and locomotor activity were not significantly different between the16/6-Id-injected and the control mice. Immunohistochemistry analysis revealed an increase in astrocytes and microglial activation in the hippocampus and amygdala, in the 16/6-Id injected group compared to the control.

**Conclusions:**

Passive transfer of 16/6-Id antibodies directly into mice brain resulted in cognitive impairments and histological evidence for brain inflammation. These findings shed additional light on the diverse mosaic pathophysiology of neuropsychiatric lupus.

See related Commentary article: http://www.biomedcentral.com/1741-7015/11/91

## Background

Neuropsychiatric systemic lupus erythematosus (NPSLE) refers to a complex set of syndromes involving the central nervous system (CNS) in up to 56% of lupus patients [1-5]. Due to the varied diagnostic criteria applied to define NPSLE, the American College of Rheumatology has proposed a standard nomenclature of case definitions, reporting standards and diagnostic testing recommendations for the 19 neuropsychiatric Systemic lupus erythematosus (SLE) syndromes [6]. While some of the focal manifestations (for example, stroke) can be explained by vasculitic or thrombotic lesions, the pathogenicity of more diffuse manifestations of NPSLE (for example, cognitive impairment, depression and psychosis) remains relatively obscure. Nevertheless, studies have demonstrated the importance of various factors involved in the development of diffuse neuropsychiatric manifestations, such as the presence of autoantibodies, inflammatory mediators (for example, cytokines, matrix metalloproteinases), neuropeptides and endocrine factors [7-10]. Other factors, such as medications and primary neurologic and psychiatric disorders, may play a major role as well.

More than 20 brain specific and non-specific autoantibodies have been proposed to be involved in the mechanism of NPLSE [11], including anti-neuronal [12], anti-ribosomal–P [13,14], anti-phospholipid [15] antibodies, as well as anti NR2/anti-DNA antibodies that cross react with N-methyl-D-aspartate (NMDA) receptors [3,16]. During the last two decades, anti-DNA idiotypes were characterized, and found to play an important role in systemic lupus erythematosus and NPSLE [17]. The 16/6 idiotype (Id) antibody is a human anti-single-stranded-DNA (anti-ssDNA) monoclonal antibody (mAb) originated from a patient with cold agglutinin disease [18]. The 16/6-Id was found to be polyspecific [19], cross reacting with cytoskeletal proteins (vimentin), platelets, lymphocyte membranes, pathogens such as *Klebsiela* polysaccharides and *Mycobacterium tuberculosis* glycoproteins, brain glycolipids and tumor cells [20-22]. The presence of 16/6-Id was detected in 30% of lupus patients, and their levels were found to correlate with disease activity [23,24]. Elevated titers of 16/6-Id were also detected in NPSLE patients [25]. Deposits of 16/6-Id were found in the skin, kidney and brain tissue [21,26,27], and were found to bind human cortical brain tissue sections *ex vivo*. The presence of circulating 16/6-Id was detected in patients with other autoimmuine diseases as well (for example, polymyositis, systemic sclerosis) [28,29]. Immunization of naïve mice with the human anti-DNA 16/6-Id mAb was shown to induce experimental lupus manifested both serologically and clinically. A wide profile of mice autoantibodies (for example, mouse 16/6-Id, and antibodies against dsDNA, ssDNA, Ro, La, RNP, Sm, histones, cardiolipin and phosphatydilserine), were detected, as well as leukopenia, elevated erythrocyte sedimentation rate (ESR), proteinuria and the deposition of immunoglobulins in the kidney mesangium [30-32]. In addition, recent-preliminary data showed histological brain changes in mice with experimental SLE induced by active immunization with the 16/6-Id (A. Marom and E. Mozes, unpublished results). Therefore, we hypothesized that the 16/6-Ids have a pathogenic role in neuropsychiatric lupus. In the present study we investigated the effect of 16/6-Id on behavioral and cognitive functions, as well as on the brain pathology of naïve mice injected intra-cerebra-ventricularily (ICV) with the 16/6-Id.

## Methods

### Mice, antibody injection and experimental design

#### Mice

Three-month-old, female C3H mice were obtained from Harlan Laboratories, Jerusalem, Israel, and were housed in the animal facility at Sheba Medical Center. The mice were raised under standard conditions, 23 ± 1°C, 12-hour light cycle (6:30 am to 6:30 pm) with *ad libitum* access to food and water. The Sheba Medical Center Animal Welfare Committee approved all procedures.

#### Monoclonal 16/6-Id expressing antibodies

The human monoclonal anti–DNA antibodies were produced by a hybridoma derived from fusion of the GM4672 lymphoblastoid cell line and peripheral blood or splenic lymphocytes obtained from three lupus patients. The human mAb that bears the 16/6-Id (IgG1/k) has been characterized previously [33]. The mAb was secreted by hybridoma cells that were grown in culture and were purified by using a protein G-sepharose column (Pharmacia, Fine Chemicals, Uppsala, Sweden).

The injection process is based on a detailed protocol reported by Shoenfeld *et al.*[34]. Mice were anesthetized by intra-peritoneal (i.p.) injection of ketamine (100 mg/kg) and xylazine (20 mg/kg). The skull was carefully exposed, and a small hole was drilled with a 25-gauge needle above the right lateral ventricle (2 mm lateral to the midline and 2.5 mm posterior to the bregma). A 27-gauge needle attached to a Hamilton syringe was inserted at this point to a depth of 2 mm, where preliminary tests had confirmed accurate ICV placement by injection of dye. Then 1 μl of anti-DNA 16/6-Id mAb or control IgG was slowly infused, the needle was withdrawn and the skin over the scalp was sutured. All antibody solutions used contained 6 mg protein/ml. Each mouse received only a single injection.

#### Experimental design

Twenty-one CH3 mice were injected ICV to the right hemisphere, 11 with human 16/6-Id antibodies and 10 with commercial human IgG antibodies (control). The forced swimming test (FST) was performed at Days 16 and 23 after antibody injection, the staircase test at Days 14 and 26, the novel object recognition at Days 19 and 20 and the Y-maze test at Day 21. At Day 24, under anesthesia, a systemic perfusion was performed, and the brains were collected. Immunofluorescence staining was performed to detect markers of inflammation or neuronal degeneration (see below).

### Cognitive and behavioral tests

#### Forced swimming test

This test is based on Porsolt *et al.*‘s description [35]. Mice were placed in individual glass beakers (height 39 cm, diameter 21.7 cm) with water 15 cm deep at 25°C. On the first day, mice were placed in the cylinder for a pretest session of 15 minutes, and later were removed from the cylinder, and then returned to their home cages. Twenty-four hours later (Day 2), the mice were re-exposed to the swimming condition in a similar environment, and then subjected to a test session for six minutes. The behavioral measure scored was the duration (in seconds) of immobility, defined as the absence of escape-oriented behaviors, such as swimming, jumping, rearing, sniffing or diving, recorded during the six-minute test. A depression-like behavior was considered as an increased immobility time.

#### Staircase test

Locomotor and explorative activity was evaluated by the staircase test, as described previously by Katzav *et al.*[15]. This test analyzes locomotor and exploratory activities (stair-climbing) and anxiety (rearing). The staircase maze consisted of a polyvinyl chloride enclosure with five identical steps, 2.5 × 10 × 7.5 cm. The inner height of the walls was constant (12.5 cm) along the whole length of the staircase. The box was placed in a room with constant lighting and isolated from external noise. Each mouse was tested individually. The animal was placed on the floor of the staircase with its back to the staircase. The number of stairs climbed and the number of rears were recorded during a three-minute period. Climbing was defined as each stair on which the mouse placed all four paws; rearing was defined as each instance the mouse rose on hind legs (to sniff the air), either on the stair or against the wall. The number of stairs descended was not taken into account. Before each test, the animal was removed and the box cleaned with a diluted alcohol solution to eliminate smells.

#### Novel object recognition test

This is a visual recognition memory test based on a method described by Tordera *et al.*[36]. The apparatus, an open field box (50 × 50 × 20 cm), was constructed from plywood painted white. Three phases (habituation, training and retention) were conducted on two separate test days. Before training, mice were individually habituated by allowing them to explore the box for one hour. No data were collected at this phase. During training sessions, two identical objects were placed into the box in the northwest and southeast corners (approximately 5 cm from the walls), 20 cm away from each other (symmetrically) and then the individual animal was allowed to explore for five minutes. Exploration of an object was defined as directing the nose to the object at a distance of ≤1 cm and/or touching it with the nose; turning around or sitting near the object was not considered as exploratory behavior. The time spent in exploring each object was recorded. The animals were returned to their home cages immediately after training. During the retention test, the animals were placed back into the same box after a four-hour interval, and allowed to explore freely for five minutes. One of the familiar objects used during training was replaced by a novel object. All objects were balanced in terms of physical complexity and were emotionally neutral. The box and the objects were thoroughly cleaned by 70% alcohol before each session to avoid possible instinctive odorant cues. A preference index, a ratio of the amount of time spent exploring any one of the two items (old and new in the retention session) over the total time spent exploring both objects, was used to measure recognition memory. Individual animals demonstrating insufficient task performance were excluded from later specific statistical analyses for the following reasons: (1) non-exploration, which was defined as no objection interaction or (2) technical malfunctions during data collection.

#### Y maze test

The Y maze test was used to assess spatial memory. It was comprised of three arms, built of black Perspex. Each arm was 8 × 30 × 15 cm at an angle of 120° from the others. One arm was randomly selected as the start arm. Each mouse was placed twice in the start arm. On the first trial, lasting for five minutes, one of the other two arms was randomly chosen to be blocked whereas on the second trial, lasting for two minutes, both arms were open. The two trials were separated by a two-minute interval, during which the mouse was returned to its home cage. The time spent in each of the arms was measured. Between each trial and between each mouse, the maze was cleaned with a 70% alcohol solution and dried. Discrimination of spatial novelty was assessed by a preference index [37]: time in the new arm - time old arm/time in the new arm + time in the old arm, assessing spatial memory. The mouse is expected to recognize the old arm as old and spend more time in the new arm.

### Immunofluorescence staining

#### Brain perfusion and fixation

The mice were anesthetized by an i.p. injection of ketamine (100 mg/kg) and xylazine (20 mg/kg) and sacrificed by transcardiac perfusion with phosphate buffered saline (PBS) followed by perfusion with 4% paraformaldehyde (PFA, Sigma-Aldrich Israel Ltd., Rehovot Israel) in phosphate buffer (PO4, pH 7.4). After perfusion, the brain was quickly removed and fixed overnight in 4% PFA (in PO4, pH 7.4) at 4°C. On the following day, the brain was cryoprotected by immersion in 30% sucrose in 0.1M PO4 (pH 7.4) for 24 to 48 hours at 4°C before brain cutting.

#### Brain cutting and preservation

Frozen coronal sections (30 to 50 μm) were cut on a sliding microtome (Leica Microsystems GmbH, Wetzlar, Germany), collected serially and kept in a cryoprotectant at −20°C until staining. Staining was performed as follows. Six mice (three IgG control and three 16/6 Id) were used for immunohistochemistry. Brain sections were stained free-floating, incubated with the first antibodies overnight at 4°C. The slices were then washed in PBS + 0.1% Triton X-100, and incubated at room temperature for one hour with the corresponding fluorescent chromogens-conjugated secondary antibody. Sections were stained for specific antigens with antibodies against activated microglia (anti-Iba1, pAb, Abcam, Cambridge, UK) and astrocytes (anti-GFAP mAb, Dako, Carpinteria, CA, USA). Counter staining was performed with Hoechst (Sigma-Aldrich Israel Ltd., Rehovot Israel).

### Statistical analysis

Results are expressed as the mean ± SEM. The differences in mean for average immobility time in the FST, the staircase test parameters (number of rearing and stair-climbing events), novel object recognition and Y-maze tests were evaluated by *T*-test. Significant results were determined as *P* <0.05.

## Results

### Cognitive and behavioral performance

**Figure 1:**
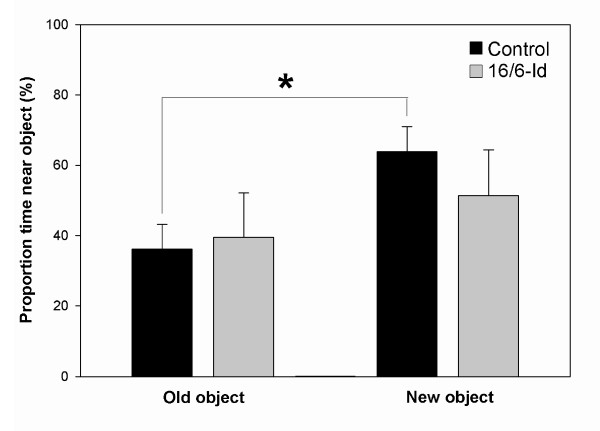
**16/6-Id injected mice displayed impaired performance in the novel object recognition test.** Results are presented as the proportion of time spent near the old and new objects by the 16/6-Id (gray bars) and IgG control (black bars) injected mice. The control mice (IgG) significantly preferred the new object (64% vs. 36% for the proportion time near the new vs. old objects respectively; *P* = 0.01), while the 16/6-Id injected mice had no significant preference to either objects (56% vs. 44% new vs. old; *P* = 0.5). Results presented as mean ± SEM. * Statistically significant (*P* <0.05).

**Figure 2:**
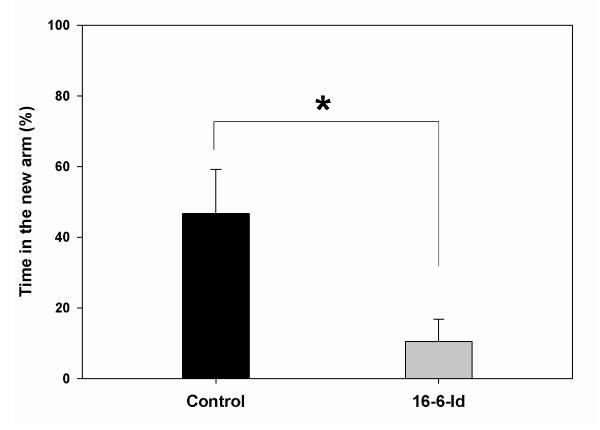
**16/6-Id injected mice displayed impaired spatial memory in the Y-maze test.** Results are presented as the proportion of time (mean ± SEM) spent in the new arm introduced by the 16/6-Id (gray bars) and IgG control (black bars) injected mice. In the figure it is shown that the control group (IgG injected) spent more time in the new lane as compared to the 16/6 injected group. They have recognized the old lane as known and preferred exploring the new lane, which means that their spatial memory is conserved. There was a significant difference in additional time spent in the new lane between the 16/6 and IgG group (0.46 vs. 0.09, *P* = 0.02 respectively). * Statistically significant (*P* <0.05).

The results of cognitive performance in the novel object recognition test are presented as the proportion time spent near objects (new and old) in both groups (Figure 1). There was a significant preference for attention to the new object in the control group (64% time spent near the new object compared to 36% time spent near the old object, *P* = 0.012), while no difference in the preference was seen in the mice injected with 16/6-Id (56% vs. 44% time spent near the new object vs. old object, *P* = 0.655). This suggests a specific visual recognition memory impairment in the 16/6-Id mice. Similarly, cognitive performance in the Y-maze test is presented as a preference index for new (additional percent time spent in the novel arm) in both groups (Figure 2). The control IgG mice spent 46% additional time in the new lane while the mice injected with 16/6-Id spent 9% additional time in the new lane (*P* = 0.015 by *t*-test).

In the forced swimming test there was no significant difference between 16/6-Id injected and control mice in depression-like behavior at Days 16 and 24 after injection. Average immobility times of the control mice vs. 16/6-Id injected mice were 117.6 ± 65.9 vs. 160 ± 72.8 (*P* = 0.159 by *t*-test) and 182.5 ± 45.4 vs. 205.7 ± 42.7 sec (*P* = 0.238 by *t*-test) on Days 16 and 24, respectively.

In the staircase test, there was no significant difference between the average rearing and stair-climbing counts, among mice from control-IgG vs. 16/6-Id (23.7 ± 2.6 vs. 21.8 ± 2.5 rearings, and 24.5 ± 2.3 vs. 16.5 ± 4.4 stair-climbing events, respectively, *P* >0.016). The results also did not change from Day 14 to 26.

### Brain pathology

**Figure 3:**
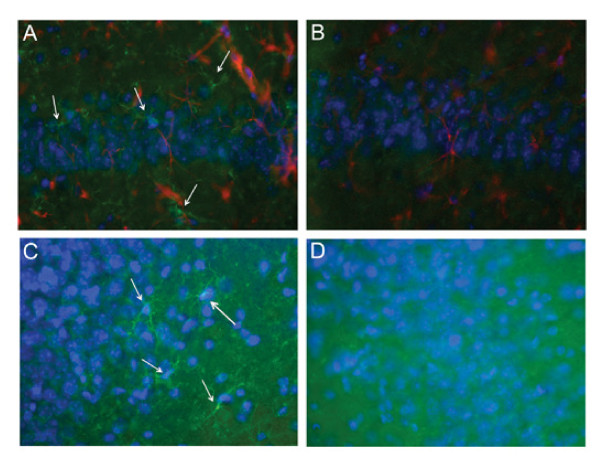
**Increased brain inflammation (activated microglia) in 16/6-Id mice in the hippocampal regions (CA1, CA3).** Staining of activated microglia (green, white arrows) was more prominent in the 16/6-Id injected mice brains (**A, C**) compared to control mice brains (**B, D**) in the hippocampal regions CA1 (**A, B**) and CA3 (**C, D**). Hoechst nucleus staining – blue, GFAP staining – red. Magnification ×40.

**Figure 4:**
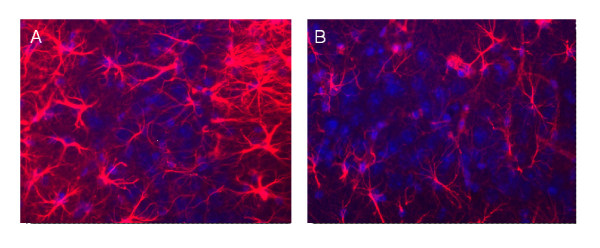
**Increased brain inflammation (astrocyes) in 16/6-Id mice in the hippocampal region (CA3).** Staining of astrocytes (red) in the hippocampal CA3 region was more prominent in the 16/6-Id injected mice brains (**A**) compared to control mice injected with commercial IgG (**B**). Hoechst nucleus staining - blue. Magnification ×40.

Brain sections were stained for activated microglia and astrocytes (as markers for inflammation). The 16/6-Id injected mice demonstrated increased microglial activation (Iba-1 staining), at the hippocampus (CA1, CA3, dentate gyrus, stratum radiatum) as well as the amygdala, compared to IgG control (Figure 3). The difference in microglial activation staining was not seen in the neucortex and piriform cortex, between 16/6-Id and control-IgG mice. Increased staining for astrocytes (GFAP staining) was also noted in the CA3 hippocampal region in the 16/6-Id injected mice compared to controls (Figure 4).

## Discussion

In the present study we have observed that passive transfer of 16/6-Id antibodies directly to mice brains resulted in a selective cognitive impairment, expressed as visual recognition and spatial memory deficits. Depressive behavior (FST) and locomotor activity (staircase test) were not altered in the 16/6-Id injected mice, when compared to the control group. Our findings suggest that 16/6-Id antibodies may have a role in the pathogenesis of cognitive impairment observed in some patients with SLE [8].

Immunostaining of brain sections from both groups revealed increased presence of activated microglia and astrocytes, in the hippocampal region of the 16/6-Id injected mice, compared to the controls. The hippocampus has an important function in memory processing, therefore, its damage by an inflammatory processes may affect cognitive performance in the 16/6-Id injected mice. Astrocytes in steady state conditions are mainly responsible for biochemical support and several other chemical roles such as maintenance of extracellular ion balance. However, in special situations, astrocytes may increase in number as an inflammatory reaction aimed for scaring and repairing CNS tissue. Microglia serve as scavengers and are activated in an inflammatory reaction. The presence of more astrocytes (gliosis) or the activation of microglia in brain tissue can implicate an inflammatory state. Our hypothesis regarding the pathogenesis of 16/6-Id antibodies induced-brain impairment includes several mechanisms: 1) Neuronal degeneration may be caused by direct or indirect injury to hippocampal area. For example, recently Berry *et al*. demonstrated that anti-ATP synthase autoantibodies, purified from Alzheimer’s disease patients, can lead to cognitive impairment and hippocampal neuron apoptosis in naïve mice [38]. Other neurotoxic autoantibodies, such as anti-phospholipid and anti-ribosomal P antibodies, were shown to penetrate living cells and cause functional cellular injury and apoptosis by inhibiting protein synthesis [39,40]. 2) Neuronal function modification. 16/6-Id antibodies may recognize and bind antigens expressed on neurons of the hippocampus and may affect brain cells by alter signaling, cell function and neurotransmitter pathways [41]. 3) Brain inflammation. Injection of 16/6-Id antibodies may lead to brain inflammation involving activation of microglia and astrocytes, and the production of pro-inflammatory cytokines. This inflammatory response can disrupt the blood–brain barrier, facilitating entry into the brain by inflammatory factors, including circulating cells of the immune system, cytokines, immune-complex mediated small vessel inflammation, and complement components. The inflammatory reaction may induce cognitive changes observed in the injected mice.

We have extensively studied the pathogenesis of different autoantibodies and their influence on the brain. Injection of anti-ribosomal-P antibodies ICV to naïve mice resulted in depressive-like behavior in these mice [42,43]. In another study, we found that injection of antiphospholipid syndrome patients with antibodies induced memory deficits and hyperactivity [15,44]. This suggests that a certain antibody is linked with each specific disease manifestation. The presence of numerous autoantibodies, at least 174 in SLE and 20 in NPSLE, which might have a role in the mechanism of the disease were reported during the past years [11,45]. This may explain the diversity of 19 neuropsychiatric manifestations which can be demonstrated in more than 50% of SLE patients [46]. We propose a hypothesis, that in NPSLE patients different manifestations are the result of an interplay among various auto-antibodies and genetic and environmental factors. For this process to occur, auto-antibodies produced in the body must be able to cross the blood–brain barrier (BBB). It is presumed that the BBB can become transiently “unlocked” following an inflammatory insult, an immune complex damage or exposure to infectious endotoxins (for example, lipopolysaccharide, LPS), allowing antibody penetration. In addition, different auto-antibodies may attach to different epitopes, expressed unevenly in different brain areas or neuronal networks. In the studies of Diamond *et al*., anti-DNA antibodies which can cross-react with the NR2 - anti-NMDA receptor were found in the sera, CSF and brains of SLE patients [16,47]. These antibodies were shown to alter brain cell function and to mediate apoptotic death *in vivo* and *in vitro*[16,47]. In their experiments, the BBB was breached temporarily by injection with LPS to imitate an infection [48], while others used noradrenalin to imitate a stressful condition; both conditions were implicated in triggering disease flare-ups in SLE and NPSLE patients. The studies of Diamond *et al.* added another layer to the current understandings regarding the role of different auto-antibodies in the pathogenesis of NPSLE. Another technique to bypass the BBB was used by us in several experiments. In the ICV technique, antibodies were injected directly into the lateral ventricle in the mouse brain, allowing antibody dispersal throughout the brain tissue. In our previous studies, an experimental NPSLE was induced by passive transfer of anti-ribosomal-P antibodies directly to mice brains [43]. The intra-cerebra-ventricularly injected mice exhibited a depression-like behavior, not associated with motor or cognitive deficits, and was significantly attenuated by prolong treatment with an anti-depressant (fluoxetine), but not with anti-psychotic drug (haloperidol). Interestingly, the anti-ribosomal-P antibody specifically stained neurons which are related to limbic and olfactory brain areas: the hippocampus, cingulate cortex and the primary olfactory piriform cortex [43]. The depressed mice also exhibited a decreased smell threshold capability [42], as well as olfactory and limbic imaging alterations, when manganese-enhanced-magnetic resonance imaging (MRI) was performed [49].

Another issue of this puzzle was stressed almost two decades ago when the importance of the idiotypic network in the induction of various autoimmune diseases was acknowledged [22,50]. One proposed mechanism of action of the 16 /6-Id is *via* the idiotypic network, in which injection of human anti-DNA 16/6-Id mAbs induces the generation of anti-Id, and anti-anti-Id, and so on. The production of 16/6-Id was found to be induced also by several infectious agents (for example, *Klebsiella pneumonia*[51,52] and *Mycobacterium tuberculosis*[53]); this could point to the role of infections in initiating the disease in a genetically susceptible individual [54].

The finding, that 16/6-Id antibodies were detected in other autoimmune diseases, such as PM/DM and scleroderma without them expressing central nervous symptoms is interesting. Perhaps, in some diseases (for example, SLE) a variety of systemic factors enable the altering of BBB permeability. These factors may include other circulating antibodies, inflammatory elements, as well as vasogenic agents, growth factors and free radicals. This phenomenon is not unusual in the autoimmunity field, for instance, anti-Ro antibodies are associated with myositis or sub-acute skin manifestations in some SLE patients and not in Sjogren patients.

The current finding, that the 16/6-Id is related to spatial novelty and visual recognition memory impairments in mice, may attest for immune-mediated damage to brain areas relevant for these functions. There is a wide agreement that spatial long-term memory and object recognition is dependent on the functioning of the hippocampal region [55]. Taken together, these concepts may promote the idea for a treatment for NPSLE via blocking or inhibiting the 16/6-Id. This can be done perhaps by treatment with intravenous gamma-globulin, which harbors anti-idiotypic antibodies itself, and has shown some efficacy in the treatment of NPSLE patients [56]. Other therapeutic means may involve the utilization of inhibitory peptides based on the complementarity determining region of anti-DNA antibodies. Indeed, such a peptide was shown to be effective in animal models and in a limited number of lupus patients [57-59].

## Conclusions

Passive transfer of anti-DNA 16/6-idiotype directly to mice brains resulted in cognitive impairment, supported by cognitive testing impairments, and changes in brain histological analysis. Therefore, the 16/6-idiotype may have a role in cognitive decline, as well as other neuropsychiatric manifestations, which are found in lupus patients.

## Abbreviations

anti-ssDNA: Anti-single-stranded-DNA; BBB: Blood–brain barrier; CNS: Central nervous system; FST: Forced swimming test; ICV: Intra-cerebra-ventricularly; i.p: Intra-peritoneal; LPS: Lipopolysaccharide; mAb: Monoclonal antibody; MRI: Magnetic resonance imaging; NMDA: N-methyl-D-aspartate; NPSLE: Neuropsychiatric systemic lupus erythematosus; PBS: Phosphate buffered saline; PFA: Paraformaldehyde; SLE: Systemic lupus erythematosus; 16/6-Id: 16/6-idiotype

## Competing interests

The authors declare that they have no competing interests.

## Authors’ contributions

SK participated in the immunohistochemical and behavioral studies and drafted the manuscript. AK participated in the immunohistochemical and behavioral studies and helped to draft the manuscript. MTA participated in the immunohistochemical studies and performed the statistical analysis. MLR and YZ participated in the behavioral studies. NAL participated in the behavioral studies and helped to draft the manuscript. MB participated in the design and helped to draft the manuscript. JMA participated in the design and coordination of the study. EM and JC participated in the design and coordination of the study and helped to draft the manuscript. YS conceived of the study, participated in its design and coordination, and helped to draft the manuscript. All authors read and approved the final manuscript.

## Pre-publication history

The pre-publication history for this paper can be accessed here:

http://www.biomedcentral.com/1741-7015/11/90/prepub
